# Leveraging a Dual‐Focused Growth Mindset to Boost Employee Resilience and Work Well‐Being: Evidence From a Two‐Wave Survey and an Intervention Study

**DOI:** 10.1002/smi.70093

**Published:** 2025-07-29

**Authors:** Oi‐ling Siu, Yaqi Yang, Aimei Li, Huatian Wang, Ting Kin Ng

**Affiliations:** ^1^ Department of Psychology Lingnan University Hong Kong China; ^2^ Party School of Yunnan Committee of CPC Yunnan Academy of Governance Kunming China; ^3^ School of Management Jinan University Guangzhou China

**Keywords:** growth mindset, intervention, resilience, work well‐being

## Abstract

The post‐pandemic era, coupled with the rising adoption of AI chatbots and robotics, introduces significant new challenges for employee work well‐being. Thus, it is important to investigate underlying mechanisms about how employees can develop mindsets to promote well‐being at work. This study examines how a dual‐focused growth mindset—comprising a growth mindset about the self (the belief in the ability to develop personal abilities) and a growth mindset about work (the belief in the capacity to optimise work conditions)—can enhance employee work well‐being through resilience. In a two‐wave survey involving 606 full‐time employees in China (Study 1), we found that both mindsets were associated with lower levels of mental ill‐health symptoms (one dimension of work well‐being) by increasing personal resilience. Notably, the effect of a growth mindset about the self (but not about work) on personal resilience was stronger when individuals perceived a high (vs. low) level of work stress. In Study 2, a quasi‐experimental design with 85 participants in an intervention group and 66 in a control group demonstrated that a growth mindset intervention effectively enhanced dual growth mindsets, leading to improved well‐being, including job satisfaction and individual flourishing. A serial mediation analysis confirmed that resilience mediated the relationship between the self‐growth mindset (not work‐growth mindset) and employee flourishing. Theoretical and practical implications were discussed.

## 1 Introduction

Work well‐being refers to employee overall affective experience and functioning at wok (Siu et al. 2005; Wang, Ding, et al. 2022). The rapid evolution of artificial intelligence (AI), digitalisation, and automation is reshaping the nature of work and substantially influencing employees' work well‐being (Parker and Grote 2022). To navigate this fast‐changing and increasingly complex work environment, scholars and practitioners emphasise the importance of equipping individuals with a growth mindset—a positive belief that personal attributes can be developed through effort (C. Dweck 2016). Research has shown that a growth mindset is essential for coping with ongoing organizational changes, technological disruptions, and evolving career demands (Berg et al. 2023; Demerouti et al. 2019).

While previous research has demonstrated that a growth mindset can enhance learning (Parada and Verlhiac 2021), creativity (Han and Stieha 2020), and career development (Burnette et al. 2022), much of this work conceptualises growth mindset as a single‐dimensional construct, primarily focussing on growth in the self, such as personal ability and intelligence (Burnette et al. 2022; Ka and Lam 2020; Sheffler et al. 2023). However, today's workplace demands are more than self‐development alone. Employees must also believe they can shape and improve their external work conditions—a belief that they can proactively adjust tasks, relationships, and responsibilities in the workplace to better fit their strengths and goals. Without this belief, even highly motivated individuals may feel stuck in rigid roles, leading to disengagement and burnout (Bakker et al. 2023). This work‐focused growth mindset aligns with the job crafting literature (Tims et al. 2012), which emphasises the value of proactively shaping one's work environment to sustain performance and well‐being.

We argue that both mindsets—self‐growth and work‐growth—are needed in tandem. A self‐growth mindset enables employees to stay motivated and open to learning (C. Dweck 2016), while a work‐growth mindset empowers them to actively shape their work conditions to meet personal and organizational goals (Berg et al. 2023). Relying on only one mindset may result in an imbalance—either passive acceptance of unfavourable conditions or ineffective efforts to adapt without personal capabilities and readiness. Together, these orientations form a dual‐growth mindset—a belief system encompassing both the capacity for personal development and the agency to optimise one's work environment (Berg et al. 2023). Such a dual‐focused mindset may be particularly powerful in modern workplace, where fixed routines are rare, and changes in team structures, collaboration patterns, and strategies are frequent (Karoly 1993; McGrath et al. 2000; Ocasio 1997).

This leads to our central question: how and under what conditions can dual‐growth mindsets (comprising both self‐growth and work‐growth mindsets) facilitate employee work well‐being, and how to develop an effective intervention to cultivate dual‐growth mindsets among employees?

**FIGURE 1:**
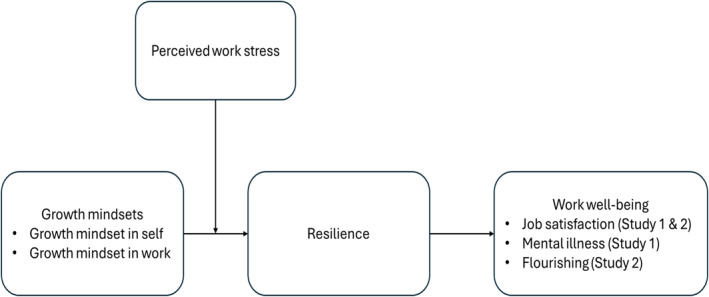
Conceptual model (Study 1 and 2).

To address these questions, we conduct two studies. In Study 1, drawing upon the conservation of resources (COR) theory (Hobfoll et al. 2018) and using a two‐wave, time lagged survey, we examine how dual‐growth mindsets facilitate employee work well‐being via increasing resilience, and whether this relationship is more salient under high (vs. low) stressful work environments. In Study 2, we use the feedback learning theory (Thurlings et al. 2013) and the job demands‐resources (JD‐R) framework (Demerouti and Bakker 2023) to develop a dual‐growth mindset intervention to train employees with the dual‐focused growth mindset. Using the field experimental approach, we assess whether the dual‐growth mindset is trainable and whether it can foster actual improvements in employee work well‐being, which can provide more causality effects (See conceptual model in Figure 1).

Our study contributes to existing literature in several ways. First, we enrich the growth mindset studies (Burnette et al. 2022, 2023; Chen et al. 2019; Parada and Verlhiac 2021) by extending the construct to the workplace and conceptualising it as a dual‐dimensional belief system. We conceptualise and empirically investigate two distinct dimensions of growth mindsets in the workplace—self‐growth mindset and work‐growth mindset—highlighting how these dual aspects of growth mindsets serve as vital facilitators, enabling the development of other important personal resources (e.g., resilience) and enhancing employee work well‐being. In doing so, we further contribute to the COR theory (Hobfoll et al. 2018) by illustrating how specific cognitive beliefs are not just helpful attitudes, but also powerful starting points in the resource accumulation process. Additionally, we as answer the call from new propositions on the JD‐R theory in times of crisis (Demerouti and Bakker 2023), providing insights into how personal resources can be activated and the health and well‐being enhancement process positively influenced by growth mindsets.

Second, we contribute to work design studies (Grant and Parker 2009; Oldham and Fried 2016) by suggesting how growth mindsets, particularly work‐growth mindsets, can help employees actively design healthier work conditions, especially when experiencing high work stress. Work‐growth mindset can represent the flexibility of job designs, as individuals holding this mindset are situationally aware of their job conditions and willing to take proactive steps to create positive changes (Berg et al. 2023). This bottom‐up, agentic approach to managing one's own well‐being complements top‐down, manager‐driven changes. We further enrich the SMART work design model (Parker and Knight 2024) and work design in the digital age research (Demerouti 2020; Parker and Grote 2022) by identifying mindset as a critical cognitive enabler of proactive job redesign.

Third, by developing an effective growth mindset intervention, we demonstrate that the dual‐growth mindset is trainable and can lead to substantial improvements in employee work well‐being. Previous growth mindset intervention studies have shown limited effectiveness, with mixed results on employee well‐being outcomes (see a review, Burnette et al. 2022). Recent studies by Sheffler et al. (2023) and Schleider and Weisz (2018) found that growth mindset interventions failed to improve cognitive functioning or reduce anxiety among participants. Our study provides new evidence that a targeted dual‐growth mindset can be effective. By integrating growth mindsets as a precursor to behavioural change, our intervention complements existing job crafting and work redesign interventions (Demerouti et al. 2019; Lambert et al. 2022) and contributes to well‐being oriented human resource management literature (Guest 2017; Luu 2019). We underscore that when job redesign intervention is complemented with dual growth mindsets, employees can better equip themselves to customise their redesigned jobs by using increased personal resources and accrue greater benefits over time.

### 1.1 Literature Review and Development of Hypotheses

#### 1.1.1 Conservation of Resources (COR) Theory

The current study draws on COR theory (Hobfoll 2002) to develop its hypotheses. According to COR theory, individuals actively strive to obtain, retain, protect, and build valuable resources (Hobfoll 2002). Those who possess abundant resources typically demonstrate enhanced resilience, gain energy, and cope more effectively with stressful events (Halbesleben et al. 2009; Hobfoll et al. 2018). COR theory defines resources broadly as valuable entities that individuals seek to acquire and preserve (Hobfoll 2002). These resources can be categorised into three primary types: personal resources, social resources, and structural resources (Hobfoll et al. 2018; ten Brummelhuis and Bakker 2012). Personal resources encompass an individual's internal abilities, including skills, knowledge, self‐esteem, and positive psychological traits (Xanthopoulou et al. 2007). Social resources consist of external resources obtained through interpersonal networks and relationships, such as social support, mentoring, and positive social interactions (Hobfoll et al. 2018; ten Brummelhuis and Bakker 2012). Structural resources are tangible external assets accessible to individuals, including financial resources, equipment, and facilities (Tims et al. 2012).

Following this theoretical logic, a dual‐focused growth mindset—defined as the belief that individuals can continuously develop their *personal* skills and attributes (growth in the self) and proactively adjust or redesign their *work conditions* (growth in work) (Berg et al. 2023)—could be classified as a crucial personal resource. Specifically, a dual‐focused growth mindset can foster individual resilience by enhancing individuals' capacity to view work challenges as personal developmental opportunities and work redesign opportunities (Berg et al. 2023; Wrzesniewski and Dutton 2001).

### 1.2 Study 1: How and When a Dual‐Focused Growth Mindset Facilitates Work Well‐Being

A dual‐focused growth mindset comprises two interrelated components: growth in the self and growth in work. Specifically, growth mindset about the self involves believing that one's abilities, intelligence, and traits are not fixed, but can be developed and improved (Zarrinabadi et al. 2021). Growth mindset about work, on the other hand, embodies the belief that people can be agentic to change and optimise their work conditions, such as work tasks and relationships (Berg et al. 2023) (see Table 1 for more clarifications on the conceptual distinctions between self‐focused growth mindset and work‐focused growth mindset). A self‐focused growth mindset helps employees develop and strengthen personal resources, such as persistence, learning, and self‐efficacy (C. S. Dweck 2013). Employees who believe that their abilities, intelligence, and competencies can grow through effort and learning are more likely to persevere through failures and actively seek self‐improvement (Burnette, et al. 2020). By contrast, a work‐focused growth mindset enables employees to *manage and optimise their work‐related resources* by encouraging proactive bottom‐up strategies. Employees who believe they can modify their tasks, work relationships, and job conditions tend to engage in proactive efforts to improve their work experiences and personal resources (Parker et al. 2010; Wrzesniewski and Dutton 2001). Thus, a work‐focused growth mindset involves a psychological empowerment process and a tendency towards job crafting actions, where employees perceive greater control over their work environment and have stronger intentions to reshape their job resources and demands (Bakker et al. 2023).

**TABLE 1:** The conceptual distinctions between self‐focused growth mindset and work‐focused growth mindset.

	Self‐focused growth mindset	Work‐focused growth mindset
Definition	The belief that personal abilities, intelligence, and competencies can develop through effort and learning.	The belief that work conditions, job roles, and job challenges can be modified and improved through proactive effort.
Resource focus (COR theory)	Focuses on developing *personal resources* (e.g., persistence, learning, self‐efficacy).	Focuses on managing and optimising *work‐related resources* (e.g., job tasks, work environment, relationships).
Key mechanism	Enhances persistence and continuous learning, helping employees sustain and build psychological resources.	Encourages proactive bottom‐up strategies and self‐empowerment, allowing employees to modify and optimise their work environment.

Employee work well‐being refers to employees' overall affective experience and their functioning in the workplace (Siu et al. 2005; Wang, Ding, et al. 2022). Many scholars have agreed that high work well‐being means employees have a pleasurable or positive emotional state resulting from the appraisal of their job or job experiences (Judge et al. 2000), and also feel less stress‐induced mental ill health, such as panic attacks and constant tiredness (Siu et al. 2005). Based on the conceptualisation of employee dual‐growth mindsets (Berg et al. 2023; C. S. Dweck 2013) and previous evidence (Chen et al. 2019; Y. Zhao et al. 2018), we argue that a dual‐focused growth mindset can positively link to employee work well‐being. A dual‐growth mindset enables employees to perceive tasks, relations, and circumstances in a positive manner, and also act in a more self‐motivated way (Rattan and Dweck 2018). This positivity can bring in higher psychological resources, which results in a generally positive evaluation of the job and good condition of mental health (Luthans and Youssef‐Morgan 2017; Newman et al. 2014). Previous studies found that an individual's growth mindset can enhance his/her learning satisfaction (Chen et al. 2019; Y. Zhao et al. 2018), work engagement (Zeng et al. 2019), and overall well‐being (Cutumisu et al. 2018; Li et al. 2021).

### 1.3 The Mediating Role of Resilience

A remaining question is by what mechanism a dual‐growth mindset can link to higher employee work well‐being. Based on COR theory (Hobfoll et al. 2018), we argue that an important mechanism is that a dual‐growth mindset can help individuals build and conserve personal resources, which in turn, enhance work well‐being (e.g., affective experience and functioning). Following this logic, we propose and examine the mediating role of resilience which is also one construct of psychological capital (Siu 2013).

Resilience is described as the human ability to adapt in the face of various life stressors (e.g., adversity, hardship, and other ongoing stressors) (Newman et al. 2014). From a process perspective, other scholars have framed resilience as a process to harness resources to sustain well‐being (Southwick et al. 2014). Schwarz (2018) highlighted two important processes in resilience: positive adaptation and adversity coping. Luthans and Youssef‐Morgan (2017) considered resilience as one of the four important psychological resources. According to the COR theory and the literature on individual proactivity (Crant 2000), those with a dual‐growth mindset—that is, a growth mindset about both the self and their work—are more likely to believe that both their personal abilities and external work environment can be improved through effort. A self‐growth mindset involves the belief that one's personal qualities and capabilities can develop over time. This belief strengthens resilience by promoting adaptive self‐regulation and persistence in the face of personal or situational challenges. Individuals with a strong self‐growth mindset are more likely to view setbacks as learning opportunities, which helps them recover emotionally and stay engaged despite adversity (Rattan and Dweck 2018). A work‐growth mindset, by contrast, involves the belief that one's job role, tasks, or work environment can be shaped and improved through effort and proactive behaviour. This mindset encourages employees to take the initiative, seek resources, and modify aspects of their work to better fit their goals and well‐being. As a result, they are more capable of navigating workplace stressors and maintaining performance under pressure (Chen et al. 2019). To summarise, we argue that a dual‐growth mindset may activate a resilient affective‐behavioural process by which employees can adapt to stressful events, recover from setbacks, and maintain continuity (Hobfoll et al. 2003).

Subsequently, we argue that resilience can link to a higher level of work well‐being. The COR theory suggests that individuals strive to use their personal resources (e.g., resilience) to cultivate better well‐being and deal with various life stressors (Siu 2013). Studies have shown that resilience was positively related to positive work experience, work‐family enrichment, and happiness in life (c.f., Choi et al. 2018; Siu et al. 2021; Wang et al. 2023). Evidence on mental health also suggests that resilience can protect individuals from mental ill‐health symptoms (Lo 2021), because it can facilitate cognitive reappraisal and self‐smoothing (Schroder et al. 2015). Thus, we argue that employees with high resilience can actively adapt to a demanding work environment and swiftly recover from stress‐induced mental ill health. Taken together.

Hypothesis 1 Growth mindsets about (a) the self and (b) work will be positively related to resilience, which in turn, will be positively related to employee work well‐being.

### 1.4 The Moderating Role of Perceived Work Stress

We further argue that having a dual‐growth mindset may be more effective when perceived work stress is high. Based on the COR theory (Hobfoll et al. 2018), individuals strive to acquire and protect resources that they value, especially when these resources are threatened. The gain paradox principle with COR theory posits that resource gains are more impactful and meaningful in the context of high resource loss or threat—such as during periods of elevated work stress (Hobfoll et al. 2018). In a highly stressful work situation, people holding a dual‐growth mindset—believing both in their capacity for personal development and in their ability to shape their work environment—may be especially well‐equipped to generate resource gains. Under such conditions, individuals are more motivated to invest personal resources to cope with challenges, and those with a dual‐growth mindset are more likely to see stressors as opportunities for learning, growth and proactive changes (Berg et al. 2023). Thus, a dual‐growth mindset becomes a more critical and valuable psychological resource in high‐stress environments compared to low‐stress ones, enabling individuals to acquire new resources and protect against further resource loss. Empirical evidence supports the notion that personal resources are more helpful in stressful conditions. For example, previous studies have shown that self‐efficacy and optimism were more positively related to well‐being when work stress was high (Kalimo et al. 2002). S. Zhao et al. (2023) found a negative correlation between a growth mindset and perceived stress in educational settings. Extending this logic, we argue that a dual‐growth mindset acts as a personal resource whose influence on resilience and well‐being is particularly pronounced when employees are exposed to high levels of work stress. Thus, we hypothesise:

Hypothesis 2 Perceived work stress will moderate the effects of growth mindsets about (a) the self and (b) work on resilience, such that this positive effect of growth mindsets will be stronger when the level of perceived work stress is higher.

Hypothesis 3 Perceived work stress will moderate the effects of growth mindsets about (a) the self and (b) work on work well‐being through resilience, such that this indirect effect of growth mindsets will be stronger when the level of perceived work stress is higher.

### 1.5 Study 1: A Two‐Wave, Time‐Lagged Survey

#### 1.5.1 Participants and Procedures

Study 1 aimed to examine H1, H2 and H3. We recruited participants from the Greater Bay Area (GBA) of mainland China. We sent out our surveys in two waves with a time interval of 6 weeks. Informed consent was obtained from participants before they filled in the questionnaire each time. In the first wave, we received 1990 valid responses. We ask participants to create an identification code, so that we can match it with the second‐wave survey. In the second wave, we only obtained 606 responses. Thus, we finally yielded 606 eligible employees who completed two‐wave surveys. Among the participants, 59.2% of them were male. 66.8% had a bachelor degree or above. 41.1% had management position in the current organization.

### 1.6 Measures

We measured IV and the moderator at T1, the mediator at T1 and T2, and DV at T1 and T2. We followed the back‐translation procedure to translate the items into Chinese.

*Growth mindset* (T1) was measured by the growth mindset scale (C. S. Dweck et al. 1995), on a six‐point Likert scale (1 = strongly disagree, 6 = strongly agree). Two dimensions were included (four items for each dimension): *growth mindset about the self* (e.g., no matter how my current abilities are, I can change it; Cronbach's *α* = 0.82), and *growth mindset about work* (e.g., no matter how my current work conditions are, I can change it; Cronbach's *α* = 0.82).

*Perceived work stress* (T1) was measured with two items using the scale by Liu et al. (2010). An example item was “I perceive that I am under a lot of pressure”, ranging from 1 (strongly disagree) to 6 (strongly agree). Cronbach's *α* = 0.83.

*Resilience* (T1 and T2) was measured with three items adapted by Siu et al. (2005), (2009). An example item was: I can recover in the face of difficulties at work. A seven‐point Likert scale (1 = strongly disagree, 7 = strongly agree) was used. Cronbach's *α* = 0.89.

#### 1.6.1 Work Well‐Being (T1 and T2)

Employee work well‐being was measured by two dimensions (Siu et al. 2005): job satisfaction and mental ill‐health symptoms. Job satisfaction measures the positive aspect of work well‐being while mental ill‐health symptoms measure the negative symptoms of well‐being. A higher level of employee work well‐being is indicated by higher job satisfaction and lower mental ill‐health symptoms in the workplace (see, Siu et al. 2005). Job satisfaction was measured with two items: “In general, I like my job.” and “All in all, I am satisfied with my job.” The Cronbach's alpha was 0.85 at T1 and 0.87 at T2. Mental ill‐health symptoms were measured with six items (e.g., I feel constant tiredness). The Cronbach's alpha was 0.85 at T1 and 0.88 at T2. All the items were rated on a six‐point Likert scale (1 = strongly disagree, 6 = strongly agree).

#### 1.6.2 Control Variables

We controlled age, gender, marital status, educational level as these demographic variables may influence the level of growth mindset and work well‐being. We also controlled for workload, as it may influence the level of resilience. We used the scale by Spector (1998) with five items. An example item was “How often does your job require you to work very hard? ”, with five‐point Likert scale (1 = less than once per month or never, 5 = several times per day). Cronbach's *α* = 0.86. Finally, we controlled for resilience at T1 and work well‐being (i.e., job satisfaction and mental ill‐health symptoms) at T1.

### 1.7 Data Analysis

We performed the structural equation modeling (SEM) analysis using SmartPLS 4.0, in which we compared several different measurement models and chose the best‐fitting one, as well as drew and evaluated the structural model accordingly. To examine the mediation effect and moderated mediation effect, we used the Process approach with 5000 bootstrapping and provided 95% bias‐corrected confidence intervals.

## 2 Results

### 2.1 Preliminary Analysis

Due to the page limits, we presented the correlation results among the studied variables in Table 2. Regarding the measurement model (i.e., the confirmatory factor analysis approach), we tested several models and selected the best‐fitting one. Due to page limits, we put the results in the supplementary document (see Table A). To conclude, the best fitting model was a six‐factor model (two factors of the growth mindset, resilience, perceived work stress, and two factors of work well‐being): χ2 (438) = 917.79; CFI = 0.93; TLI = 0.92; SRMR = 0.05; RMSEA = 0.04, which was significantly better than other alternative models. We also performed the unmeasured latent method factor analysis (Podsakoff et al. 2011) using Mplus (also see, Wang, Rispens, et al. 2022) to examine the common method bias (CMB). We found that the potential “method” factor shared a variance of 0.04 (i.e., 3.57% of shared variance). Thus, we concluded that CMB might not be a serious issue in the current study.

**TABLE 2:** Means, S.D., and correlations among studied variables (Study 1).

										
		*M*	*SD*	1	2	3	4	5	6	7	8	9	10	11
1	Growth mindset about the self T1	3.59	0.94											
2	Growth mindset about work T1	4.35	0.82	0.50**										
3	Resilience T1	4.52	0.85	0.36**	0.42**									
4	Perceived work stress T1	2.28	0.78	−0.13**	−0.03	−0.21**								
5	Job satisfaction T1	4.03	1.18	0.25**	0.27**	0.47**	−0.31**							
6	Mental ill‐health symptoms T1	2.83	0.96	−0.06	−0.25**	−0.25**	0.49**	−0.31**						
7	Growth mindset about the self T2	3.56	0.99											
8	Growth mindset about work T2	4.25	0.82							0.47**				
9	Resilience T2	4.51	0.83							0.29**	0.37**			
10	Job satisfaction T2	4.03	1.18							0.30**	0.31**	0.42**		
11	Mental ill‐health symptoms T1	2.81	0.99							−0.04	−0.12**	−0.25**	−0.28**	

*Note: N* = 606.

* refers to *p* < .05.

** refers to *p* < .01.

### 2.2 Hypothesis Testing

**FIGURE 2:**
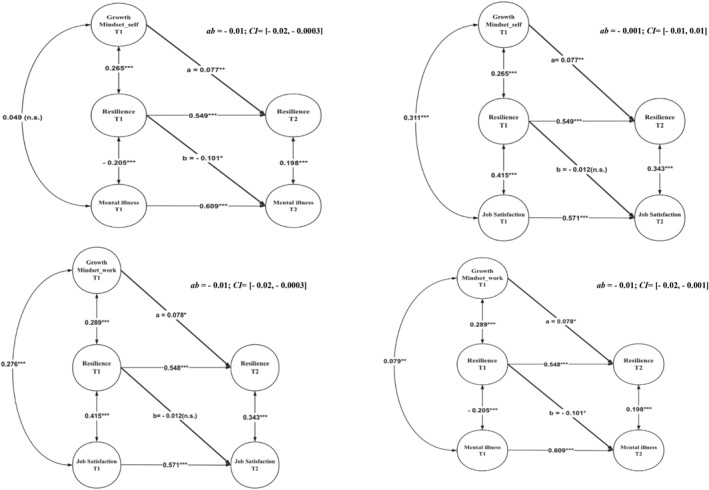
Results on the mediation effects of growth mindset about the self and in work on mental ill‐health symptoms and job satisfaction through resilience (Study 1).

We drew a figure to present the results of the main effects of the growth mindset (see Figure 2): growth mindset about the self was significantly related to a lower level of mental ill‐health symptoms via resilience (*ab* = −0.01, CI = [−0.02, −0.0003]). Growth mindset about work was significantly related to a lower level of mental ill‐health symptoms via resilience (*ab* = −0.01, CI = [−0.02, −0.001]). However, growth mindsets about the self and in work were not significantly related to job satisfaction via resilience (*ab* = −0.01, CI = [−0.01, 0.01]; *ab* = −0.001, CI = [−0.01, 0.01]). Therefore, H1 was only partially supported.

**FIGURE 3:**
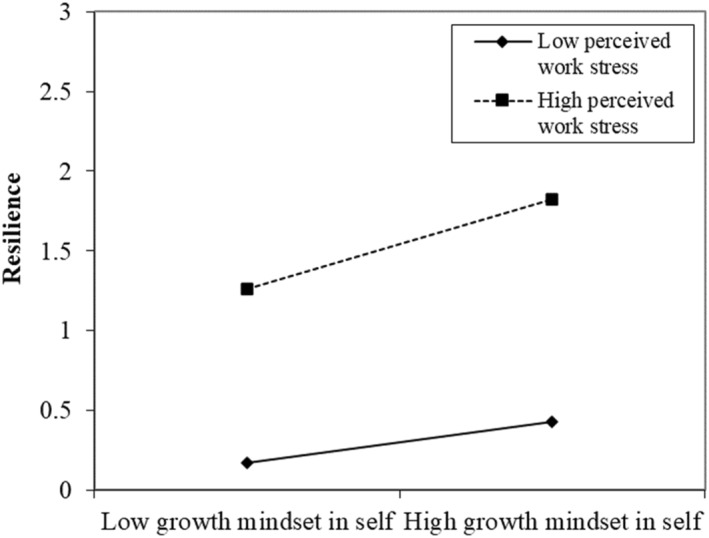
The two‐way interaction effect between growth mindset about the self and perceived work stress on resilience (Study 1).

Regarding the moderating effect of perceived work stress, our results showed the significant two‐way interaction terms between growth mindset about the self and perceived work stress on resilience (*b* = 0.07, *p* < 0.05). We further conducted the simple slope test, which showed that growth mindset about the self was more positively related to resilience when the level of perceived work stress was high (*b* = 0.29, *p* < 0.01), compared to when it was low (*b* = 0.13, *p* < 0.001). We plotted a two‐way interaction figure (See Figure 3). However, we did not find a significant two‐way interaction between growth mindset about work and perceived work stress on resilience (*b* = 0.04, *p* = 0.33). Therefore, only H2a was supported.

We assessed the conditional indirect effects using PROCESS, but the results were not significant. There was no significant moderated mediation of perceived work stress on the relationship between growth mindset about the self and job satisfaction via resilience (*b* = 0.08, CI = [−0.02, 0.04]). Similarly, the relationship between growth mindset about work and job satisfaction via resilience was also not significant (*b* = 0.002, CI = [−0.03, 0.03]). Additionally, the moderated mediation for perceived work stress on the relationship between growth mindset about the self and mental ill‐health symptoms via resilience was not significant (*b* = −0.004, CI = [−0.03, 0.02]), nor was it significant for growth mindset about work and mental ill‐health symptoms (*b* = −0.001, CI = [−0.02, 0.02]). Therefore, H3 was rejected.

### 2.3 Discussion of Study 1

In Study 1, we used a two‐wave survey design to examine the beneficial role of dual‐growth mindsets on employee resilience, which in turn, facilitated their job satisfaction and reduced the potential of mental ill‐health symptoms. We found that growth mindsets about the self and work can significantly relate a lower level of mental ill‐health symptoms via increasing personal resilience. But we did not find evidence for job satisfaction. Further, our results showed that growth mindset about the self (not in work) can particularly buffer the negative influence of perceived work stress. While insightful, Study 1 had some limitations. First, we only measured growth mindsets at T1, and thus we cannot draw a cross lagged panel model to improve the causality. Second, we did not find the effect of dual‐growth mindset on job satisfaction (one affective aspect of employee work well‐being). We also did not measure the functioning aspect of work well‐being (e.g., flourishing). Therefore, Study 2 will adopt a quasi‐experimental design to address the above issues but also to examine whether dual‐growth mindsets are trainable.

### 2.4 Study 2: Stimulating Dual‐Focused Growth Mindsets via an Intervention

In addition to understanding the role of employee dual‐growth mindsets in the workplace, we believe it is crucial to develop an intervention that can train employees to cultivate such mindsets in their daily work setting. This can provide more causal effects of dual‐growth mindsets on employee resilience and their work well‐being. To design our intervention, we used the feedback learning theory (Thurlings et al. 2013) and the JD‐R framework (Demerouti and Bakker 2023)—two theories widely used in the design of workplace cognition, task, and relationship training programs (van Wingerden et al. 2017; Eklöf and Hagberg 2006).

The intervention includes the workshop and self‐practice assignments. The workshop aims to raise participants' awareness of the importance of dual‐growth mindsets and increase their intention to apply these mindsets at work. Drawing on feedback learning theory (Thurlings et al. 2013), we taught participants how to develop *self‐growth mindset* by learning from others' experiences and feedback and self‐reflecting upon their personal abilities and strengths. Based on JD‐R framework (Demerouti and Bakker 2023), we taught participants the main characteristics of the workplace (i.e., job resources and job demands) and how to get access to job resources by seeking and learning from others' experiences and feedback, which can particularly nurture participants' *work‐growth mindset*. The assignment session then sought to further strengthen participants' awareness and application of dual‐growth mindsets, as well as increase their perceived control over applying these mindsets in their daily work. Participants were asked to set SMART (specific, measurable, achievable, relevant, and time‐bound) goals (Locke and Latham 2016) to practice developing dual‐growth mindsets through feedback‐learning and job resource‐seeking processes. Based on the JD‐R model (Bakker et al. 2023; Demerouti and Bakker 2023) and the job crafting literature (Lichtenthaler and Fischbach, 2019; Zhang and Parker 2019), job crafting can be categorised into promotion‐focused job crafting (e.g., seeking job resources and challenges) and prevention‐focused job crafting (e.g., reducing job demands). Aligning with this research, dual‐focused growth mindsets are more promoted‐focused. Given this promotion‐oriented perspective, our intervention specifically encouraged participants to focus on seeking job resources rather than reducing job demands. The full details of our intervention design and content are presented in Table 3. We suspect this intervention will be effective: Instructing participants to use and make sense of external feedback can activate their learning processes and self‐reflection on personal growth, stimulating a self‐growth mindset. Likewise, heightening participants' awareness of job demands and resources can encourage them to be more agentic in adjusting their work conditions, thereby cultivating a work‐growth mindset.

**TABLE 3:** Overview of the training and assignments (Study 2).

Intervention	Module	Content
Training Workshop	1. Growth Mindset *Topic: What is a growth mindset?*	• The training introduced the background, concepts, research, and implications of both self‐growth mindset and work‐focused growth mindset. It aimed to explain their impact on individuals from childhood to adulthood and raise awareness of dual‐growth mindsets in the workplace, emphasising their potential to foster personal and professional development.
		• Materials: A 6‐min introduction video about growth mindsets.
	2. Neural Mechanism *Topic: What is neural plasticity?*	• The training explained the neural mechanisms of the self‐growth mindset by illustrating how the brain functions, highlighting the concept of neural plasticity and the strengthening or weakening of neural pathways. It presented evidence that intelligence is malleable and can be developed through effort, emphasising that challenging tasks and learning new skills are the best exercises for the brain, fostering growth and adaptability.
		• Materials: A 2‐min video introduced neural plasticity. A 3‐min video showed your brain is like a muscle.
	*3.* Feedback Learning *Topic: Is feedback a useful job resource? How to learn from failure and success?*	• The job demands‐resources theory highlights feedback as a critical job resource (Schaufeli and Bakker 2004). Seeking feedback is a proactive way to manage job demands, and fostering the belief that individuals can seek feedback from others can strengthen work‐focused growth mindsets (Yang 2024). To support this, we explained reinforcement learning theory and feedback learning methods using the example of AlphaGo (Silver et al. 2017). This demonstrated how learning from feedback is highly applicable to real‐life and workplace scenarios, where standard answers or guidance may be lacking, and mentors or instructors are often unavailable. This approach underscores the importance of feedback in driving growth and adaptability in dynamic environments.
		• Materials: A 3‐min clip of the documentary *Alpha Go* introduced how a machine learns from feedback. A 5‐min go competition video showed the difference between humans and machines when facing failure and success.
	4. Mindset reshaping Exercise *Topic: How to apply it in the workplace?*	• Using the examples from real life and work settings to instruct how to better deal with negative feedback (mistakes, burnout, rejection, failure etc.) and learn from positive feedback by developing our self‐ and work‐growth mindsets. We designed choice questions and language‐rewriting exercises, contextualised within the workplace setting, to help participants recognise growth mindset traits and shift fixed language patterns into growth‐oriented ones.
		• Materials: Four sets of multiple‐choice questions designed to identify the characteristics of a dual‐focused growth mindset. Ten sentence rewriting exercises designed to reshape mindset.
Self‐Assignment (After daily work, choose one of these three assignments to complete. Last for five working days.)	**Morning survey (8:30 a.m.)** **Question (goal setting):** What do you want to change about yourself (self‐growth mindset) or your work (work‐growth mindset) today? **Method: Choose one feedback learning method to help yourself and your work.** 1. Self‐feedback learning mindset exercise (learn from your own working experience) 2. Observational feedback learning mindset exercise (learn from others' working experience) 3. Social feedback learning mindset exercise (learn from the working feedback provided by your supervisor/colleagues/clients/friends/family, etc.)	
	**Evening survey (5:30 p.m.)** **Reflection Instructions** **Step 1:** Please review the feedback you received today, whether from your own past experiences (self‐feedback), observing others' experiences (observational feedback), or input from your social connections (social feedback). Briefly describe the feedback and its source. **Step 2:** Please rate your (or their) performance today in achieving your goal—whether changing yourself or improving your work conditions—on a scale from 1 to 10. **Step 3:** (1) learn from failure (if the score is less than 10): Please reflect on what difficulties or challenges have you (other) encountered in the course of the work and why did this happen? What were your (his/her) thoughts, decisions and behaviors at that time? Whether there were better solutions? How to improve? Please write down your thoughts. (2) Learn from success (if the score reaches 10): Please reflect on what were the key elements that contributed to the success of this work? What thoughts and behaviors of yours (their) have contributed to the achievement? How to learn and transfer this successful experience to your (their) other work? **Step 4:** For your next attempt, predict your performance (1–10) and write one growth mindset statement to encourage yourself.	

*Note:* In the assignment, Step 1 and 2 were designed to facilitate employees to reflect and evaluate the working experience, which is the learning material for mindset exercise. Step 3 served to apply growth mindset and learn from the working feedback, including failures and successes. Step 4 for creating a prediction for better performance in the future.

Hypothesis 4 Participants in the intervention group will report a higher improvement in the growth mindset about (a) the self and (b) work than those in the control group.

Following the reasoning of hypothesis 1, we argue that the growth mindset intervention can further enhance employee work well‐being. This is because when employees hold dual‐growth mindsets, they will be able to shape perceptions, effort beliefs, and goals. As a result, employees can exhibit more positive psychological, cognitive, and behavioural processes and consequences (Rattan and Ozgumus 2019). Employees can gain more positive experience and functioning in the workplace. Thus, we hypothesise.

Hypothesis 5 Participants joining in the intervention will report a higher improvement in the growth mindsets about (a) the self and (b) work, which in turn, report a higher improvement in work well‐being (the mediation effect of the intervention).

Finally, we argue that the growth mindset intervention can enable participants to exhibit higher resilience, which in turn, enhance well‐being. We believe that by joining in the intervention, participants will engage in deeper self‐reflection on their personal abilities, traits, and work conditions. Through the feedback‐learning and job resources‐seeking processes, they can develop a stronger sense of agency over workplace challenges (Marks 1998). This heightened sense of personal agency and the ability to proactively shape work circumstances can instill greater resilience in the participants (Berg et al. 2023; Rattan and Dweck 2018). Following the COR theory, when individuals are able to effectively protect and build up their personal resources, such as resilience, they are more likely to experience positive emotions and function better in the work context (Hobfoll et al. 2018). Thus:

Hypothesis 6 Participants joining in the intervention will report higher improvements in the of growth mindsets about (a) the self and (b) work, which in turn, will report a higher improvement in resilience and subsequently a higher improvement in work well‐being (the serial mediation effect of the intervention).

### 2.5 Study 2: A Field Quasi‐Experiment

#### 2.5.1 Participants

Study 2 aimed to examine H4, H5, and H6. We recruited participants through the cooperation with institutions in mainland China and Hong Kong. The interventions were conducted separately for different institutions, including government education bureau, private kindergartens, state‐owned enterprises in mainland China and universities in Hong Kong. Participants with full‐time employment or working experience were eligible to register. They were required to attend a face‐to‐face training workshop, self‐practise session and self‐reflection session after the workshop over five working days.

Before we recruited participants, we conducted a priori power analysis to determine our sample size. According to the calculation (2 groups combined with 2 time point measurements at the 95% confidence level), a sample size of 54 for each group is needed. Considering the dropout rate of 30% in prior research, we decided to recruit 78 participants for both intervention group and control group.

**FIGURE 4:**
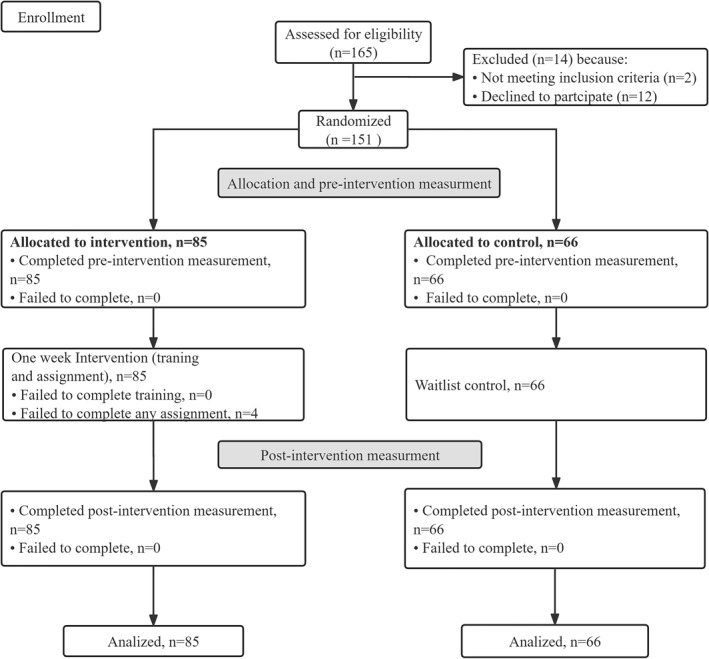
Flow diagram (Study 2).

The flow diagram of the participant recruitment was shown in Figure 4. Participants were randomized to the intervention group and the control group. Finally, 151 participants filled in both pre‐ and post‐surveys, with a number of 85 participants in the intervention group and 66 in the control group. The intervention group consisted of 71 women and 14 men; 82.4% were younger than 40 years old; 96.5% had completed a higher vocational education or university education, 49.4% of them held a supervisory position. The control group consisted of 45 women and 21 men; 90.9% were younger than 40 years old; 95.5% had completed a higher vocational education or university education, 65.2% of them held a supervisory role.

### 2.6 Intervention Design and Procedure

The training was designed to be completed in 1 week, ensuring a focused yet impactful experience. On Day 1, participants engaged in a 1.5‐h face‐to‐face workshop exploring growth mindset concepts, neural mechanisms, feedback learning, and mindset‐reshaping exercises. The session introduced dual‐growth mindsets to heighten their awareness of self and work growth. It emphasised developing abilities through effort, leveraging feedback for improvement, and challenging limiting beliefs through practical activities. The workshop aimed to equip attendees with tools to embrace challenges, learn from failures, and foster a positive mindset. Reference to Table 3 provided further details on specific exercises and frameworks. Overall, it served as an introduction to growth mindset principles, promoting both personal and professional development.

In the training, we first introduced both self‐focused growth mindset (e.g., believing in the malleability of intelligence, abilities, or personality) and work‐focused growth mindset (e.g., believing in the capacity to optimise work conditions, tasks, and relationships). The second part focused on self‐growth mindset, explaining how neural plasticity transforms weak pathways into strong ones, making the brain malleable and enhancing learning. The third part emphasised work‐growth mindset, using AI's feedback‐learning process as a metaphor to encourage participants to extend self‐growth principles to their work. Finally, the dual‐growth mindset section included tailored choice questions and language‐rewriting exercises for both self and work contexts, helping participants apply these concepts to their personal and professional lives.

From Day 2 to Day 6, participants engaged in a 5‐day workplace assignment to apply dual‐growth mindset principles (Yang 2024). Each day included two sessions: goal‐setting and feedback learning. At 8:30 a.m., participants set a goal by answering, *“What do you want to change about yourself (*e.g. *belief in your abilities) or your work (*e.g. *belief in your agency to change work conditions) today?”* They then chose a feedback method—self‐feedback, observational feedback, or social feedback—to seek input throughout the day. Grounded in goal‐setting theory (Locke and Latham 2016), this process motivates behaviour by aligning actions with internal intentions, fostering feedback‐seeking behaviour.

At 5:30 p.m., participants were asked to complete a reflective survey. The daily reflection component in our intervention served as a self‐regulation tool to help participants track their experiences, reinforce learning, and enhance their awareness of mindset shifts (Kitsantas and Zimmerman, 2006). What we asked participants to do is to document their reflections on challenges, insights, and actions taken to optimise their work. The survey followed four steps: (1) reviewing the feedback received, (2) rating their performance on a scale of 1–10, which created a prediction error (if below 10) or reward (if 10), (3) reflecting on reasons and solutions, and (4) re‐rating for future attempts, encouraging self‐growth or work improvement. Research has highlighted the role of feedback learning in shaping mindsets, making this structured reflection a key tool for growth (Yang 2024; Huang et al. 2025). This approach aligns with prior job crafting interventions (Dubbelt et al. 2019; Pekaar and Demerouti 2021; Wang et al. 2024), where participants are encouraged to record how they adjust job demands and resources to foster self‐directed learning. By engaging in this reflective process, we aimed to reinforce participants' internal learning, enhance self‐awareness, and promote continuous improvement in both personal and professional contexts.

To mitigate demand characteristics bias, the post‐test survey was administered 1 week after the intervention. Additionally, a follow‐up occupational mental health report was provided 3 months later, which included a detailed analysis of outcomes and actionable suggestions tailored to each participant. This two‐tiered approach—combining immediate practices with long‐term support—reinforces the training's effectiveness and demonstrates its lasting impact on employee well‐being.

### 2.7 Measures

We measured the focal constructs in both a pre‐test questionnaire (T0) and a post‐test questionnaire (T1).

*Intervention* variable was coded as the dummy variable. The intervention group was coded as 1, while the control group was coded as 0. Thus, Group × Time was seen as the independent variable to examine the effectiveness of the intervention.

*Growth mindset* was measured using the same scale in Study 1. Cronbach's *α* for growth mindset about the self was 0.87 at T0, and 0.96 at T1. Cronbach's *α* for growth mindset about work was 0.86 at T0, and 0.93 at T1.

*Resilience* was measured with three items by Siu et al. (2005). Cronbach's *α* = 0.88 at T0, and 0.81 at T1.

#### 2.7.1 Work Well‐Being

We measured job satisfaction and flourishing in Study 2. We measured flourishing instead of mental ill‐health symptoms because the focal organization required us to particularly examine employees' flourishing, as it reflects the functioning aspect of employee work well‐being. Employee flourishing refers to employees' optimal functioning at work, characterised by high levels of psychological well‐being, meaning, engagement, positive relationships, and accomplishment (Diener et al. 2010). We also encountered some ethical considerations when we planned to measure mental ill‐health symptoms of employees in the focal organization. The organizational leader felt it inappropriate to measure mental related variables due to the work characteristics of employees. Thus, in Study 2, we believe that measuring flourishing can provide additional insights of employee work well‐being, in addition to mental ill‐health symptoms. Flourishing was measured with eight items (e.g., I am engaged and interested in my daily activities). A seven‐point Likert scale was used from 1 = strongly disagree to 7 = strongly agree. Cronbach's alpha = 0.90 at T0 and 0.93 at T1. Cronbach's alpha for job satisfaction = 0.76 at T0 and 0.87 at T1.

### 2.8 Data Analysis

We used the repeated‐measures GLM to examine the effectiveness of our intervention. Partial eta‐squared (η^2^) values indicate the effect sizes. As the data follow a repeated‐measures design with measurement points (level 1) nested within individuals (level 2), we used the MLmed approach to examine the mediation effects.

## 3 Results

We presented the correlation results among studied variables in Table 4. We presented ANOVAs results for both intervention group and control group in Table 5. The results showed that growth mindsets were significantly increased after the intervention (Time × group: *F* = 46.4, *p* < 0.001 for growth mindset about the self; Time × group: *F* = 18.91, *p* < 0.001 for growth mindset about work). Thus, hypothesis 4 was supported, indicating that our intervention was effective.

**TABLE 4:** Correlations among the study variables (Study 2).

	1	2	3	4	5	6	7	8	9	10
*Pre‐measure (T0)*										
1. Growth mindset about the self										
2. Growth mindset about work	0.43**									
3. Resilience	0.27**	0.30**								
4. Job satisfaction	0.21**	0.28**	0.51**							
5. Flourishing	0.20*	0.26**	0.66**	0.55**						
*Post‐measure (T1)*										
6. Growth mindset about the self	0.51**	0.39**	0.22**	0.18*	0.14					
7. Growth mindset about work	0.27**	0.59**	0.28**	0.30**	0.22**	0.73**				
8. Resilience	0.23**	0.43**	0.78**	0.45**	0.60**	0.32**	0.44**			
9. Job satisfaction	0.21**	0.27**	0.54**	0.73**	0.45**	0.37**	0.47**	0.54**		
10. Flourishing	0.12	0.29**	0.60**	0.49**	0.69**	0.32**	0.46**	0.68**	0.60**	

*Note: N =* 151.

* *p* < .05.

** *p* < .01.

**TABLE 5:** Mean scores, S.D., *t*‐tests, and repeated measures ANOVAs for the studied variables (Study 2).

	Experimental group				Control group				Repeated measures ANOVA time *X* group		
Variable	*M*	SD	*T*‐test	*p*	*M*	SD	*T*‐test	*p*	*F*	*p*	η^2^
Growth mindset about the self pre	2.74	0.74			3.11	0.77					
Growth mindset about the self post	3.44	0.73	−9.90	**< 0.001**	3.08	0.81	0.36	0.72	46.4	**<. 001**	0.24
Growth mindset about work pre	3.54	0.54			3.70	0.66					
Growth mindset about work post	3.70	0.64	−2.71	**0.01**	3.48	0.61	3.41	**< 0.001**	18.91	**< 0.001**	0.11
Resilience pre	2.27	0.46			2.31	0.48					
Resilience post	2.31	0.47	−1.05	0.30	2.26	0.47	1.31	0.19	2.80	0.10	0.02
Job satisfaction pre	1.85	0.31			1.85	0.35					
Job satisfaction post	1.87	0.31	−0.69	0.49	1.81	0.41	1.73	0.09	3.08	0.08	0.02
Flourishing pre	3.31	0.57			3.46	0.64					
Flourishing post	3.35	0.61	−0.84	0.40	3.43	0.69	0.48	0.63	0.84	0.36	0.07

*Note: N* = 85 in the intervention group, *N* = 66 in the control group. *T*‐test refers to Paired‐sample *t*‐tests. *η* ^2^ = [0.01–0.06], small effects, [0.06–0.14], medium effects, *η* ^2^ ≥ 0.14, large effects. The bold values indicate significant results (*p* < 0.05).

Although the ANOVAs result did not show significant mean differences for the resilience and two aspects of work well‐being, it did not mean that the intervention was not effective for these outcomes. One of the reasons might be that the ANOVAs, in nature, compared the mean differences among groups. Therefore, as suggested by intervention scholars (Dubbelt et al. 2019), we further conducted the multilevel regression analysis to examine whether the *indirect effects* of intervention on work well‐being. Using the MLmed approach, the results showed (see Table 6) that the intervention significantly employee job satisfaction (*b* = 0.65, CI = [0.23, 1.11]; *b* = 0.19, CI = [0.04, 0.40]) and flourishing (*b* = 1.77, CI = [0.94, 2.68]; *b* = 0.39, CI = [0.08, 0.81]) through increasing growth mindsets. These results can support hypothesis 5, indicating that the growth mindset intervention *indirectly* improved employee work well‐being.

**TABLE 6:** Results of the multilevel indirect effects of the intervention using MLmed (*N* = 151 participants, and 302 data points) (Study 2).

Control for age, gender, marital status, and educational level	*b*	*se*	*p*‐value	LLCI	ULCI
*Indirect effects*					
Time × group –> growth mindset about the self –> resilience	0.53	0.27	0.05	0.01	1.09
Time × group –> growth mindset about work –> resilience	0.04	0.08	0.57	−0.10	0.22
Time × group –> growth mindset about the self –> job satisfaction	0.65	0.22	0.004	0.23	1.11
Time × group –> growth mindset about work –> job satisfaction	0.19	0.09	0.04	0.04	0.40
Time × group –> growth mindset about the self –> flourishing	1.77	0.44	< 0.001	0.94	2.68
Time × group –> growth mindset about work –> flourishing	0.39	0.19	0.04	0.08	0.81

To test hypothesis 6, we conducted a serial mediation analysis using PROCESS by computing a difference score of studied variables (c.f., Tellhed et al. 2019). Our results (in Table 7) showed that the intervention group significantly led to changes of flourishing through changes of growth mindset about the self and then changes of resilience: *b* = 0.18, CI = [0.03, 0.52]. However, for the growth mindset about work and the other aspect of employee work well‐being—job satisfaction, we did not find significant serial mediation effects. Thus, hypothesis 6 was partially supported.

**TABLE 7:** Results of the serial mediation effects of the intervention using PROCESS (Model number 6) (Study 2).

	*b*	*p*‐value	LLCI	ULCI
*Indirect effects*				
Group –> Δ growth mindset about the self –> Δ resilience –> Δ job satisfaction	0.03	0.56	−0.07	0.14
Group –> Δ growth mindset about work –> Δ resilience –> Δ job satisfaction	0.004	0.85	−0.03	0.06
Group –> Δ growth mindset about the self –> Δ resilience –> Δ flourishing	0.18	0.02	0.03	0.52
Group –> Δ growth mindset about work –> Δ resilience –> Δ flourishing	0.02	0.78	−0.11	0.17

*Note: N* = 85 in the intervention group, *N* = 66 in the control group. Bootstrapping iterations are 5000.

Abbreviations: LLCI = lower level confidence intervals; ULCI = upper level confidence intervals.

## 4 Discussion

Based on the COR theory, this study examined how dual‐growth mindsets can facilitate employee work well‐being through enhancing resilience. Two empirical studies (one survey study and one intervention study) generally confirmed our hypotheses. The results of Study 1 showed that employees' self‐growth mindset and work‐growth mindset were positively related to resilience, which in turn, linked to a lower level of mental ill‐health symptoms. The results of Study 2 confirmed the effectiveness of our designed dual‐growth mindset intervention. Those who joined the intervention reported a higher level of dual‐growth mindsets, which in turn, had a higher level of well‐being (including job satisfaction and individual flourishing). Finally, a serial mediation analysis confirmed the mediating role of resilience on the relationship between self‐growth mindset (but not work‐growth mindset) and employee flourishing. To conclude, our study provides insights into the beneficial role of dual‐growth mindsets in boosting employee work well‐being. We offer a managerial kit (a growth mindset intervention programme) to help employees and organizations to cultivate dual‐growth mindsets.

### 4.1 Theoretical Contributions

First, this study adds to the growth mindset literature (Burnette et al. 2022, 2023; Chen et al. 2019; Parada and Verlhiac 2021) by applying the role of a dual‐growth mindset in the workplace and examining its impact on employee work well‐being through resilience. Previous studies on the growth mindset have mainly examined the growth mindset in the school setting where students or adolescents equip themselves with a growth mindset to achieve more study outcomes (Cutumisu et al. 2018). While C. Dweck (2016) highlighted that a growth mindset is an important cognitive resource for working employees, previous studies have primarily considered the growth mindset as solely concerning changes in the self, such as abilities, intelligence, and traits, as well as associated outcomes such as self‐motivation and cognitive gains (Y. Zhao et al. 2018). Few studies have considered that in the workplace, individuals cannot focus solely on changes in the self, but must also be agentic toward their work conditions and actively shape their jobs. Building on the work of Berg et al. (2023) regarding the concept of work‐growth mindset, we conceptualise and test a dual‐growth mindset that integrates both self‐growth and work‐growth orientations. We emphasise that work and self‐growth mindsets are related but distinct (see the CFA results): self‐growth mindset is a broad, internally driven belief in personal development, rooted in a generalised learning orientation (Yeager and Dweck 2020), while work‐growth mindset is domain‐specific and focuses on skill development and performance improvement in professional settings (Berg et al. 2023). Although these mindsets may reinforce each other, their cognitive framing and focus could differ. Our findings extend the work of Berg et al. (2023) by showing that a dual‐growth mindset fosters resilience, which in turn improves work well‐being—particularly under high perceived work stress. This implies that such dual‐growth mindsets can help employees adapt to dynamic and uncertain work environments by fostering both internal growth and agentic engagement with their job conditions.

Accordingly, these findings can contribute to the COR theory (Hobfoll et al. 2018) by suggesting that dual‐growth mindsets are not just personality traits but resource‐generating beliefs that enable individuals proactively build their resource reservoir. Study 1, in particular, highlights that dual‐growth mindsets are associated with higher well‐being via increased resilience. This aligns with COR's resource caravan concept (Hobfoll et al. 2018), suggesting that certain personal resources (e.g., mindsets) can create gain spirals by enabling the acquisition and protection of other key resources, like resilience. Furthermore, the stronger effects under high work stress support the gain paradox principle: resource gains are more psychologically meaningful and impactful in situations of high threat. Thus, our study not only applies COR theory but also extends it by showing how specific cognitive beliefs, such as dual‐growth mindsets, function as upstream resources that initiate gain processes, particularly under stressful work environments.

Second, our study enriches the work design studies (Grant and Parker 2009; Oldham and Fried 2016) by highlighting an important mindset‐level factor—a dual‐focused growth mindset. Work design studies have confirmed the important role of individual proactivity in designing their own jobs such as the bundle of tasks and relationships. For example, research has revealed the beneficial roles of job crafting (Zhang and Parker 2019), help‐seeking behaviour (Wang, Ding, et al. 2022), voice (Parker and Collins 2010), and proactive personality (Jiang 2017) in the job redesign process. The SMART work design model proposed by Parker and Knight (2024) highlights that employees need to design their work conditions to be stimulating, autonomous, mastery, relational, and tolerable. Our study adds to this line of research by incorporating the dual‐growth mindset as an important driver that enables employees to be cognitively ready for such agentic changes to their work conditions. This dual‐growth mindset may serve as a crucial first step before employees engage in other proactive behaviours, such as job crafting (Berg et al. 2023). By fostering a mindset oriented toward both personal growth and work growth, employees may be better equipped to redesign their work to align with the SMART work design principles.

Third, we developed an effective growth mindset intervention, which enriches the workplace individual‐centred intervention studies (Demerouti et al. 2019; Lambert et al. 2022). Previous workplace individual‐centred intervention studies developed successful training programs aimed at improving employee cognitions, affect, and behaviours, such as job crafting (van Wingerden et al. 2017), networking (Spurk et al. 2015), and strengths use (Meyers and van Woerkom 2017). However, interventions focused specifically on growth mindsets in the work setting have showed mixed results on employee well‐being outcomes (see a review, Burnette et al. 2022). To address this gap, our study attempted to draw upon the feedback learning theory and the JD‐R framework to develop a more effective growth mindset intervention. Our results showed that the dual‐growth mindset intervention was generally successful in improving both self‐growth and work‐growth mindsets, as well as enhancing employee work well‐being. Specifically, we found that the self‐growth mindset component increased individuals' resilience, which in turn led to greater personal flourishing. This finding can add to the JD‐R‐based intervention studies (Bakker and van Wingerden 2021; Demerouti et al. 2019; van Wingerden et al. 2017) by providing insights into how to design interventions to not only improve job resources, but also bolster personal resources, such as resilience.

### 4.2 Practical Implications

Our findings also provide several practical implications. First, both studies confirmed the beneficial role of dual‐growth mindsets in the work setting. Therefore, we strongly suggest that employees should develop and make use of a dual‐growth mindset to deal with work hassles and challenges. Our study showed that actively shaping the dual‐focused growth mindset could increase one's resilience level, which in turn, was negatively related to employee mental ill‐health symptoms at work, and positively related to job satisfaction and individual flourishing. Thus, when employees feel threats about their well‐being at work, holding a dual‐growth mindset may be the first step to sustaining work well‐being.

To ensure employees can be equipped with dual‐growth mindsets, organizations should cultivate a work culture and climate that encourages and supports the development of such mindsets. Employees should be provided with ample learning opportunities and constructive feedback, allowing them to feel capable of addressing different work challenges and difficulties (Berg et al. 2023). Additionally, organizations should consider giving employees more autonomy and allocating moderate job demands. Autonomy can increase employees' perceived control over their jobs and unlock the potential of their work abilities, while moderate job demands can sustain employees' motivation level and prevent negative psychological consequences, such as burnout and exhaustion (Bakker and Demerouti 2017).

Finally, our growth mindset intervention provides a useful kit for organizations to implement growth mindset training programs for their employees. Organizations and employees can use our intervention tutorial to cultivate both self‐growth and work‐growth mindsets and apply them in their daily work context. Such growth mindset training can be integrated into the organization's human resource management modules and team‐building programs (Beauchamp et al. 2017). By doing so, even those who have a lower initial level of growth mindset can benefit from the training and develop their own dual‐growth mindset.

### 4.3 Limitations and Future Directions

The findings of this study should be considered against several limitations, which represent fruitful future research directions. First, our results suggested that job satisfaction was not substantially influenced by the mediating role of resilience, with one exception: in Study 1, only work‐growth mindset was positively related to job satisfaction through resilience (*ab* = −0.01, CI = [−0.02, −0.0003]). One possible explanation is that resilience may not be the primary mechanism linking growth mindsets to job satisfaction. Other mediating factors may be at play, warranting further investigations to better understand how a dual‐focused growth mindset influences employee job satisfaction. Additionally, contextual and organizational factors—such as workplace culture, job demands, job resources, leadership support, and organizational constraints—may serve as boundary conditions moderating the impact of growth mindsets on job satisfaction. The absence of significant direct effects may be due to the omission of these factors. Therefore, future research should explore alternative mediating processes and boundary conditions to gain deeper insights into the relationship between dual‐focused growth mindsets and job satisfaction.

Second, another knowledge gap exists regarding whether one mindset (e.g., self‐growth mindset) serves as a prerequisite for the development of another (e.g., work‐growth mindset). In our current studies, both growth mindsets were measured simultaneously, preventing us from establishing a directional or causal relationship. Exploring the progression and longitudinal association over time would be an intriguing avenue for future research.

Third, while we propose that work‐growth mindset underlies beliefs regarding proactive work design, it may share conceptual similarities with other constructs in the job crafting literature, such as job crafting self‐efficacy and job crafting competencies. According to prior research, job crafting self‐efficacy is defined as an individual's beliefs about their capability to modify the demands and resources of their job to better fit their needs (Roczniewska et al. 2020); while job crafting competencies are the clusters of individual knowledge, skills, and abilities that are necessary to achieve personal objectives through effective job crafting problem‐solving, including comprehensive/simplistic heuristic information use and approach/avoidance problem‐solving skills (Bruning and Campion 2022). We argue that work‐growth mindset differs from job crafting self‐efficacy and competencies because it is a broader motivational belief about one's agency to change work conditions, rather than a specific belief in one's ability to execute job crafting (self‐efficacy) or the actual skills required to do so (competencies).^1^ However, we believe that work‐growth mindset may facilitate the development of job crafting self‐efficacy and competencies over time and that job crafting self‐efficacy and competencies may serve as additional pathways through which work‐growth mindset influences job crafting and job satisfaction. A promising direction for future research would be to examine whether cognitive readiness for work redesign (work‐focused growth mindset) fosters job crafting abilities and subsequent job crafting behaviours.

Fourth, we acknowledge some inconsistencies in the measurement of work well‐being across the two studies. In Study 1, we assessed work well‐being using two indicators: job satisfaction and mental health conditions. In Study 2, due to practical constraints, we were unable to include mental health measures and instead focused on flourishing. This suggests that future research should assess employee work well‐being using a more comprehensive and multi‐dimensional framework.

Finally, we acknowledge that our intervention is its short duration. To enhance its effectiveness and examine longer‐term effects, a more extended intervention with creative training elements would be beneficial. For example, a multi‐session intervention spanning several weeks or months could be more effective than a single‐session workshop. Additionally, digital and mobile learning tools may serve as valuable platforms to stimulate mindset shifts and proactive job redesign, particularly in the evolving era of AI. Indeed, previous workplace interventions have often begun with a workshop followed by self‐practice assignments (e.g., job crafting interventions; Oprea et al. 2019). Notably, some intervention studies have failed to demonstrate sustained long‐term effects once the intervention ended (Dubbelt et al. 2019; Pekaar and Demerouti 2021; Wang et al. 2024), which has been a point of criticism over decades (Gollwitzer and Sheeran 2006; Prochaska and DiClemente 1983; Weiss et al. 2016). Thus, we strongly recommend that future intervention studies strengthen their design and methodological rigour to generate longer‐term impacts.

## Consent

Informed consent was obtained from all individual participants included in the study.

## Conflicts of Interest

The authors declare no conflicts of interest.

## Supporting information

Supporting Information S1

## Data Availability

The data that support the findings of this study are available on request from the corresponding author. The data are not publicly available due to privacy or ethical restrictions.

## Endnotes

^1^
To illustrate these conceptual differences, we have made a summary table and included it in the supplementary materials, see Table B.

## References

[smi70093-bib-0001] Bakker, A. B. , and E. Demerouti . 2017. “Job Demands‐Resources Theory: Taking Stock and Looking Forward.” Journal of Occupational Health Psychology 22, no. 3: 273–285. 10.1037/ocp0000056. 27732008

[smi70093-bib-0002] Bakker, A. B. , E. Demerouti , and A. Sanz‐vergel . 2023. “Job Demands—Resources Theory : Ten Years Later.” Annual Review of Organizational Psychology and Organizational Behavior 10, no. 1: 25–53. 10.1146/annurev-orgpsych-120920-053933.

[smi70093-bib-0003] Bakker, A. B. , and J. van Wingerden . 2021. “Do Personal Resources and Strengths Use Increase Work Engagement? The Effects of a Training Intervention.” Journal of Occupational Health Psychology 26, no. 1: 20–30. 10.1037/ocp0000266. 33104373

[smi70093-bib-0004] Beauchamp, M. D. , K. J. Waldhauser , and A. Uk . 2017. “Team Building: Conceptual, Methodological, and Applied Considerations.” Current Opinion in Psychology 16: 114–117. 10.1016/j.copsyc.2017.02.031. 28813332

[smi70093-bib-0005] Berg, J. M. , A. Wrzesniewski , A. M. Grant , J. Kurkoski , and B. Welle . 2023. “Getting Unstuck: The Effects of Growth Mindsets About the Self and Job on Happiness at Work.” Journal of Applied Psychology 108, no. 1: 152–166. 10.1037/apl0001021. 35549284

[smi70093-bib-0006] Bruning, P. F. , and M. A. Campion . 2022. “Assessing Job Crafting Competencies to Predict Tradeoffs Between Competing Outcomes.” Human Resource Management 61, no. 1: 91–116. 10.1002/hrm.22081.

[smi70093-bib-0007] Burnette, J. L. , J. Billingsley , G. C. Banks , et al. 2022. “A Systematic Review and Meta‐Analysis of Growth Mindset Interventions: For Whom, How, and Why Might Such Interventions Work?” Psychological Bulletin 149, no. 3–4: 174–205: (Issue October). 10.1037/bul0000368. 36227318

[smi70093-bib-0008] Burnette, J. L. , L. E. Knouse , D. T. Vavra , E. O’Boyle , and M. A. Brooks . 2020. “Growth Mindsets and Psychological Distress: A Meta‐Analysis.” Clinical Psychology Review 77: 101816. 10.1016/j.cpr.2020.101816. 32163802

[smi70093-bib-0009] Burnette, J. L. , L. E. Knouse , J. Billingsley , S. Earl , J. M. Pollack , and C. L. Hoyt . 2023. “A Systematic Review of Growth Mindset Intervention Implementation Strategies.” Social and Personality Psychology Compass 17, no. 2: e12723. 10.1111/spc3.12723.

[smi70093-bib-0012] Chen, X. , G. Zeng , E. C. Chang , and H. Y. Cheung . 2019. “What Are the Potential Predictors of Psychological Capital for Chinese Primary School Teachers?” Frontiers in Education 4: 50. 10.3389/feduc.2019.00050.

[smi70093-bib-0013] Choi, Y. E. , E. Cho , H. J. Jung , and Y. W. Sohn . 2018. “Calling as a Predictor of Life Satisfaction: The Roles of Psychological Capital, Work‐Family Enrichment, and Boundary Management Strategy.” Journal of Career Assessment 26, no. 4: 567–582. 10.1177/1069072717723092.

[smi70093-bib-0015] Crant, J. M. 2000. “Proactive Behavior in Organizations.” Journal of Management 26, no. 3: 435–462. 10.1177/014920630002600304.

[smi70093-bib-0016] Cutumisu, M. , M. R. G. Brown , C. Fray , and G. M. Schmölzer . 2018. “Growth Mindset Moderates the Effect of the Neonatal Resuscitation Program on Performance in a Computer‐Based Game Training Simulation.” Frontiers in Pediatrics 6: 195. 10.3389/fped.2018.00195. 30023355 PMC6039560

[smi70093-bib-0018] Demerouti, E. 2020. “Turn Digitalization and Automation to a Job Resource.” Applied Psychology: 1–6. 10.1111/apps.12270.32836653

[smi70093-bib-0019] Demerouti, E. , and A. B. Bakker . 2023. “Job Demands‐Resources Theory in Times of Crises: New Propositions.” Organizational Psychology Review 13, no. 3: 209–236. 10.1177/20413866221135022.

[smi70093-bib-0021] Demerouti, E. , M. C. W. Peeters , and M. van den Heuvel . 2019. “Job Crafting Interventions: Do They Work and Why?” In Positive Psychological Intervention Design and Protocols for Multi‐Cultural Contexts, edited by L. E. VanZyl and S. R. Sr. , 103–125. Springer.

[smi70093-bib-0022] Diener, E. , D. Wirtz , W. Tov , et al. 2010. “New Well‐Being Measures: Short Scales to Assess Flourishing and Positive and Negative Feelings.” Social Indicators Research 97, no. 2: 143–156. 10.1007/s11205-009-9493-y.

[smi70093-bib-0023] Dubbelt, L. , E. Demerouti , and S. Rispens . 2019. “The Value of Job Crafting for Work Engagement, Task Performance, and Career Satisfaction: Longitudinal and Quasi‐Experimental Evidence.” European Journal of Work & Organizational Psychology 28, no. 3: 300–314. 10.1080/1359432x.2019.1576632.

[smi70093-bib-0024] Dweck, C. 2016. “What Having a ‘Growth Mindset’ Actually Means.” Harvard Buisness Review, Jan 13: 1–3.

[smi70093-bib-0025] Dweck, C. S. 2013. Self‐Theories : Their Role in Motivation, Personality, and Development. Psychology Press. 2130257

[smi70093-bib-0026] Dweck, C. S. , C. yue Chiu , and Y. yi Hong . 1995. “Implicit Theories and Their Role in Judgments and Reactions: A Word From Two Perspectives.” Psychological Inquiry 6, no. 4: 267–285. 10.1207/s15327965pli0604_1.

[smi70093-bib-0027] Eklöf, M. , and M. Hagberg . 2006. “Are Simple Feedback Interventions Involving Workplace Data Associated With Better Working Environment and Health? A Cluster Randomized Controlled Study Among Swedish VDU Workers.” Applied Ergonomics 37, no. 2: 201–210. 10.1016/j.apergo.2005.04.003. 15982632

[smi70093-bib-0029] Gollwitzer, P. M. , and P. Sheeran . 2006. “Implication Intentions and Goal Achievements: A Meta‐Analysis of Effects and Processes.” Advantages in Experimental Social Psychology 38, no. 2006: 69–119.

[smi70093-bib-0030] Grant, A. M. , and S. K. Parker . 2009. “Redesigning Work Design Theories: The Rise of Relational and Proactive Perspectives.” Academy of Management Annals 3, no. 1: 317–375. 10.5465/19416520903047327.

[smi70093-bib-0031] Guest, D. E. 2017. “Human Resource Management and Employee Well‐Being: Towards a New Analytic Framework.” Human Resource Management Journal 27, no. 1: 22–38. 10.1111/1748-8583.12139.

[smi70093-bib-0032] Halbesleben, J. R. B. , J. Harvey , and M. C. Bolino . 2009. “Too Engaged? A Conservation of Resources View of the Relationship Between Work Engagement and Work Interference With Family.” Journal of Applied Psychology 94, no. 6: 1452–1465. 10.1037/a0017595. 19916655

[smi70093-bib-0033] Han, S. J. , and V. Stieha . 2020. “Growth Mindset for Human Resource Development: A Scoping Review of the Literature With Recommended Interventions.” Human Resource Development Review 19, no. 3: 309–331. 10.1177/1534484320939739.

[smi70093-bib-0034] Hobfoll, S. E. 2002. “Social and Psychological Resources and Adaptation.” Review of General Psychology 6, no. 4: 307–324. 10.1037/1089-2680.6.4.307.

[smi70093-bib-0035] Hobfoll, S. E. , J. Halbesleben , J. P. Neveu , and M. Westman . 2018. “Conservation of Resources in the Organizational Context: The Reality of Resources and Their Consequences.” Annual Review of Organizational Psychology and Organizational Behavior 5, no. 1: 103–128. 10.1146/annurev-orgpsych-032117-104640.

[smi70093-bib-0036] Hobfoll, S. E. , R. J. Johnson , N. Ennis , and A. P. Jackson . 2003. “Resource Loss, Resource Gain, and Emotional Outcomes Among Inner City Women.” Journal of Personality and Social Psychology 84, no. 3: 632–643. 10.1037/0022-3514.84.3.632. 12635922

[smi70093-bib-0037] Huang, Y. , Y. Yang , and O. L. Siu . 2025. “Strategic Mindset Facilitates Social Feedback Processing and Self‐Concept Adjustment.” Cerebral Cortex 35, no. 3: bhaf061: (accepted paper). 10.1093/cercor/bhaf061. 40131251 PMC11934546

[smi70093-bib-0038] Jiang, Z. 2017. “Proactive Personality and Career Adaptability: The Role of Thriving at Work.” Journal of Vocational Behavior 98: 85–97. 10.1016/j.jvb.2016.10.003.

[smi70093-bib-0039] Judge, T. A. , J. E. Bono , E. A. Locke , H. B. Tippie , and T. A. Judge . 2000. “Personality and Job Satisfaction: The Mediating Role of Job Characteristics.” Journal of Applied Psychology 85, no. 2: 237–249. 10.1037/0021-9010.85.2.237. 10783540

[smi70093-bib-0040] Ka, K. , and L. Lam . 2020. “A Serial Mediation Model Testing Growth Mindset, Life Satisfaction, and Perceived Distress as Predictors of Perseverance of Effort.” Personality and Individual Differences 167, no. February: 110262. 10.1016/j.paid.2020.110262.

[smi70093-bib-0041] Kalimo, R. , K. Pahkin , and P. Mutanen . 2002. “Work and Personal Resources as Long‐Term Predictors of Well‐Being.” Stress and Health: Journal of the International Society for the Investigation of Stress 18, no. 5: 227–234. 10.1002/smi.949.

[smi70093-bib-0042] Karoly, P. 1993. “Self‐Regulation: A Systems View.” Annual Review of Psychology 44, no. 1: 23–52. 10.1146/annurev.psych.44.1.23.

[smi70093-bib-0043] Lambert, B. , B. B. Gaza , E. Trinh , and S. Asiieord . 2022. “Individual‐Centered Interventions: Identifying What, How, and Why Interventions Work in Organizational Contexts.” Academy of Management Annals 16, no. 2: 508–546. 10.5465/annals.2020.0351.

[smi70093-bib-0044] Li, M. , W. Fan , and F. T. L. Leong . 2021. “Growth Mindset of Intelligence Reduces Counterproductive Workplace Behavior: A Mediation Analysis of Occupational Stress.” International Journal of Selection and Assessment 29, no. 3–4: 519–526. 10.1111/ijsa.12347.

[smi70093-bib-0045] Liu, J. , O. L. Siu , and K. Shi . 2010. “Transformational Leadership and Employee Well‐Being: The Mediating Role of Trust in the Leader and Self‐Efficacy.” Applied Psychology 59, no. 3: 454–479. 10.1111/j.1464-0597.2009.00407.x.

[smi70093-bib-0046] Lo, A. 2021. “Using the Occupation of the Creative Arts to Promote Mental Health in Young People: Positive Mindset Creative Arts Festival.” World Federation of Occupational Therapists Bulletin 77, no. 1: 28–32. 10.1080/14473828.2020.1834256.

[smi70093-bib-0047] Locke, E. A. , and G. P. Latham . 2016. “New Directions in Goal‐Setting Theory.” Current Directions in Psychological Science 15, no. 5: 265–268. 10.1111/j.1467-8721.2006.00449.x.

[smi70093-bib-0048] Luthans, F. , and C. M. Youssef‐Morgan . 2017. “Psychological Capital: An Evidence‐Based Positive Approach.” Annual Review of Organizational Psychology and Organizational Behavior 4, no. 1: 339–366. 10.1146/annurev-orgpsych-032516-113324.

[smi70093-bib-0049] Luu, T. T. 2019. “Green Human Resource Practices and Organizational Citizenship Behavior for the Environment: The Roles of Collective Green Crafting and Environmentally Specific Servant Leadership.” Journal of Sustainable Tourism 27, no. 8: 1167–1196. 10.1080/09669582.2019.1601731.

[smi70093-bib-0050] Marks, L. I. 1998. “Deconstructing Locus of Control: Implications for Practitioners.” Journal of Counseling and Development 76, no. 3: 251–260. 10.1002/j.1556-6676.1998.tb02540.x.

[smi70093-bib-0051] McGrath, J. E. , H. Arrow , and J. L. Berdahl . 2000. “The Study of Groups: Past, Present, and Future.” Personality and Social Psychology Review 4, no. 1: 95–105. 10.1207/s15327957pspr0401_8.

[smi70093-bib-0052] Meyers, M. C. , and M. van Woerkom . 2017. “Effects of a Strengths Intervention on General and Work‐Related Well‐Being: The Mediating Role of Positive Affect.” Journal of Happiness Studies 18, no. 3: 671–689. 10.1007/s10902-016-9745-x.

[smi70093-bib-0053] Newman, A. , D. Ucbasaran , F. Zhu , and G. Hirst . 2014. “Psychological Capital: A Review and Synthesis.” Journal of Organizational Behavior 35, no. 1: S120–S138. 10.1002/job.1916.

[smi70093-bib-0054] Ocasio, W. 1997. “Towards an Attention‐Based View of the Firm.” Strategic Management Journal 18: 187–206. 10.1002/(sici)1097-0266(199707)18:1+<187::aid-smj936>3.0.co;2-k.

[smi70093-bib-0055] Oldham, G. R. , and Y. Fried . 2016. “Job Design Research and Theory: Past, Present and Future.” Organizational Behavior and Human Decision Processes 136: 20–35. 10.1016/j.obhdp.2016.05.002.

[smi70093-bib-0056] Oprea, B. T. , L. Barzin , D. Vîrgă , D. Iliescu , and A. Rusu . 2019. “Effectiveness of Job Crafting Interventions: A Meta‐Analysis and Utility Analysis.” European Journal of Work & Organizational Psychology 28, no. 6: 723–741. 10.1080/1359432x.2019.1646728.

[smi70093-bib-0057] Parada, S. , and J. F. Verlhiac . 2021. “Growth Mindset Intervention Among French University Students, and its Articulation With Proactive Coping Strategies.” Educational Psychology 42, no. 3: 354–374. 10.1080/01443410.2021.1917519.

[smi70093-bib-0058] Parker, S. K. , U. K. Bindl , and K. Strauss . 2010. “Making Things Happen: A Model of Proactive Motivation.” Journal of Management 36, no. 4: 827–856. 10.1177/0149206310363732.

[smi70093-bib-0059] Parker, S. K. , and C. G. Collins . 2010. “Taking Stock: Integrating and Differentiating Multiple Proactive Behaviors.” Journal of Management 36, no. 3: 633–662. 10.1177/0149206308321554.

[smi70093-bib-0060] Parker, S. K. , and G. Grote . 2022. “Automation, Algorithms, and Beyond: Why Work Design Matters More than Ever in a Digital World.” Applied Psychology 71, no. 4: 1171–1204. 10.1111/apps.12241.

[smi70093-bib-0061] Parker, S. K. , and C. Knight . 2024. “The SMART Model of Work Design: A Higher Order Structure to Help See the Wood From the Trees.” Human Resource Management 63, no. 2: 265–291. 10.1002/hrm.22200.

[smi70093-bib-0062] Pekaar, K. , and E. Demerouti . 2021. “Crafting for Purpose: A Daily Diary Study and Self‐Training Intervention on the Proactive Implementation of Sustainability at Work (Working Paper).” TKI Dinalog (Grant Number 2017‐2‐132TKI).

[smi70093-bib-0063] Podsakoff, P. M. , S. B. MacKenzie , and N. P. Podsakoff . 2011. “Sources of Method Bias in Social Science Research and Recommendations on How to Control It.” Annual Review of Psychology 63, no. 1: 539–569. 10.1146/annurev-psych-120710-100452. 21838546

[smi70093-bib-0064] Prochaska, J. O. , and C. C. DiClemente . 1983. “Stages and Processes of Self‐Change of Smoking: Toward an Integrative Model of Change.” Journal of Consulting and Clinical Psychology 51, no. 3: 390–395. 10.1037/0022-006x.51.3.390. 6863699

[smi70093-bib-0065] Rattan, A. , and C. S. Dweck . 2018. “What Happens After Prejudice is Confronted in the Workplace? How Mindsets Affect Minorities’ and Women’s Outlook on Future Social Relations.” Journal of Applied Psychology 103, no. 6: 676–687. 10.1037/apl0000287. 29517252

[smi70093-bib-0066] Rattan, A. , and E. Ozgumus . 2019. “Embedding Mindsets in Context: Theoretical Considerations and Opportunities for Studying Fixed‐Growth Lay Theories in the Workplace.” Research in Organizational Behavior 39, no. 2019: 100127. 10.1016/j.riob.2020.100127.

[smi70093-bib-0067] Roczniewska, M. , A. Rogala , M. Puchalska‐Kaminska , R. Cieślak , and S. Retowski . 2020. “I Believe I can Craft! Introducing Job Crafting Self‐Efficacy Scale (JCSES).” PLoS One 15, no. 8 August: 1–22. 10.1371/journal.pone.0237250. PMC7416938 32776992

[smi70093-bib-0068] Schaufeli, W. B. , and A. B. Bakker . 2004. “Job Demands, Job Resources, and Their Relationship With Burnout and Engagement: A Multi‐Sample Study.” Journal of Organizational Behavior: The International Journal of Industrial, Occupational and Organizational Psychology and Behavior 25, no. 3: 293–315. 10.1002/job.248.

[smi70093-bib-0069] Schleider, J. , and J. Weisz . 2018. “A Single‐Session Growth Mindset Intervention for Adolescent Anxiety and Depression: 9‐Month Outcomes of a Randomized Trial.” Journal of Child Psychology and Psychiatry and Allied Disciplines 59, no. 2: 160–170. 10.1111/jcpp.12811. 28921523

[smi70093-bib-0070] Schroder, H. S. , S. Dawood , M. M. Yalch , M. B. Donnellan , and J. S. Moser . 2015. “The Role of Implicit Theories in Mental Health Symptoms, Emotion Regulation, and Hypothetical Treatment Choices in College Students.” Cognitive Therapy and Research 39, no. 2: 120–139. 10.1007/s10608-014-9652-6. 35474696 PMC9037854

[smi70093-bib-0071] Schwarz, S. 2018. “Resilience in Psychology: A Critical Analysis of the Concept.” Theory & Psychology 28, no. 4: 528–541. 10.1177/0959354318783584.

[smi70093-bib-0072] Sheffler, P. , E. Kürüm , A. M. Sheen , et al. 2023. “Growth Mindset Predicts Cognitive Gains in an Older Adult Multi‐Skill Learning Intervention.” International Journal of Aging and Human Development 96, no. 4: 501–526. 10.1177/00914150221106095. 35726166 PMC10052424

[smi70093-bib-0073] Silver, D. , J. Schrittwieser , K. Simonyan , et al. 2017. “Mastering the Game of Go Without Human Knowledge.” Nature 550, no. 7676: 354–359. 10.1038/nature24270. 29052630

[smi70093-bib-0074] Siu, O. L. 2013. “Psychological Capital, Work Well‐Being, and Work‐Life Balance Among Chinese Employees: A Cross‐Lagged Analysis.” Journal of Personnel Psychology 12, no. 4: 170–181. 10.1027/1866-5888/a000092.

[smi70093-bib-0075] Siu, O. L. , C. H. Hui , D. R. Phillips , L. Lin , T. W. Wong , and K. Shi . 2009. “A Study of Resiliency Among Chinese Health Care Workers: Capacity to Cope With Workplace Stress.” Journal of Research in Personality 43, no. 5: 770–776. 10.1016/j.jrp.2009.06.008.

[smi70093-bib-0076] Siu, O. L. , Q. Kong , and T. K. Ng . 2021. “Psychological Capital and Family Satisfaction Among Employees: Do Occupational Stressors Moderate the Relationship?” International Journal of Environmental Research and Public Health 18, no. 22: 12260. 10.3390/ijerph182212260. 34832018 PMC8618752

[smi70093-bib-0077] Siu, O. L. , P. E. Spector , C. Cooper , and C. Lu . 2005. “Work Stress, Self‐Efficacy, Chinese Work Values, and Work Well‐Being in Hong Kong and Beijing.” International Journal of Stress Management 12, no. 3: 274–288. 10.1037/1072-5245.12.3.274.

[smi70093-bib-0079] Southwick, S. M. , G. A. Bonanno , A. S. Masten , C. Panter‐Brick , and R. Yehuda . 2014. “Resilience Definitions, Theory, and Challenges: Interdisciplinary Perspectives.” European Journal of Psychotraumatology 5, no. 1. 10.3402/ejpt.v5.25338. PMC4185134 25317257

[smi70093-bib-0080] Spector, P. E. 1998. “A Control Theory of the Job Stress Process.” Theories of organizational stress: 153–169. 10.1093/oso/9780198522799.003.0008.

[smi70093-bib-0081] Spurk, D. , S. Kauffeld , L. Barthauer , and N. S. R. Heinemann . 2015. “Fostering Networking Behavior, Career Planning and Optimism, and Subjective Career Success: An Intervention Study.” Journal of Vocational Behavior 87: 134–144. 10.1016/j.jvb.2014.12.007.

[smi70093-bib-0082] Tellhed, U. , D. Daukantaitė , R. E. Maddux , T. Svensson , and O. Melander . 2019. “Yogic Breathing and Mindfulness as Stress Coping Mediate Positive Health Outcomes of Yoga.” Mindfulness 10, no. 12: 2703–2715. 10.1007/s12671-019-01225-4.

[smi70093-bib-0083] ten Brummelhuis, L. L. , and A. B. Bakker . 2012. “A Resource Perspective on the Work‐Home Interface: The Work‐Home Resources Model.” American Psychologist 67, no. 7: 545–556. 10.1037/a0027974. 22506688

[smi70093-bib-0084] Thurlings, M. , M. Vermeulen , T. Bastiaens , and S. Stijnen . 2013. “Understanding Feedback: A Learning Theory Perspective.” Educational Research Review 9: 1–15. 10.1016/j.edurev.2012.11.004.

[smi70093-bib-0085] Tims, M. , A. B. Bakker , and D. Derks . 2012. “Development and Validation of the Job Crafting Scale.” Journal of Vocational Behavior 80, no. 1: 173–186. 10.1016/j.jvb.2011.05.009.

[smi70093-bib-0086] van Wingerden, J. , A. B. Bakker , and D. Derks . 2017. “Fostering Employee Well‐Being via a Job Crafting Intervention.” Journal of Vocational Behavior 100: 164–174. 10.1016/j.jvb.2017.03.008.

[smi70093-bib-0088] Wang, H. , E. Demerouti , S. Rispens , and P. van Gool . 2024. “Crafting Networks: A Self‐Training Intervention.” Journal of Vocational Behavior 149, no. October 2021: 103956. 10.1016/j.jvb.2023.103956.

[smi70093-bib-0089] Wang, H. , H. Ding , and X. Kong . 2022. “Understanding Technostress and Employee Well‐Being in Digital Work: The Roles of Work Exhaustion and Workplace Knowledge Diversity.” International Journal of Manpower 44, no. 2: 334–353. 10.1108/ijm-08-2021-0480.

[smi70093-bib-0090] Wang, H. , T. K. Ng , and O. ling Siu . 2023. “How Does Psychological Capital Lead to Better Well‐Being for Students? The Roles of Family Support and Problem‐Focused Coping.” Current Psychology 42, no. 26: 22392–22403. 10.1007/s12144-022-03339-w. PMC9209831 35756898

[smi70093-bib-0091] Wang, H. , S. Rispens , and E. Demerouti . 2022. “Boosting Creativity in Functional Diverse Work Groups: The Importance of Help‐Seeking Behavior and Openness to Experience.” European Journal of Work & Organizational Psychology 31, no. 5: 768–780. 10.1080/1359432x.2022.2047937.

[smi70093-bib-0092] Weiss, L. A. , G. J. Westerhof , and E. T. Bohlmeijer . 2016. “Can We Increase Psychological Well‐Being? The Effects of Interventions on Psychological Well‐Being: A Meta‐Analysis of Randomized Controlled Trials.” PLoS One 11, no. 6: 1–16. 10.1371/journal.pone.0158092. PMC4915721 27328124

[smi70093-bib-0093] Wrzesniewski, A. , and J. Dutton . 2001. “Crafting a Job: Revisioning Employees as Active Crafters of Their Work.” Academy of Management Journal 26, no. 2: 179–201. 10.2307/259118.

[smi70093-bib-0094] Xanthopoulou, D. , A. B. Bakker , E. Demerouti , and W. B. Schaufeli . 2007. “The Role of Personal Resources in the Job Demands‐Resources Model.” International Journal of Stress Management 14, no. 2: 121–141. 10.1037/1072-5245.14.2.121.

[smi70093-bib-0095] Yang, Y. 2024. Beyond Mindset : Investigating Neural and Psychological Mechanisms of Strategic Mindset, Growth Mindset and Feedback Learning. [PhD dissertation]. Lingnan University.

[smi70093-bib-0096] Yeager, D. S. , and C. S. Dweck . 2020. “What Can be Learned From Growth Mindset Controversies?” American Psychologist 75, no. 9: 1269–1284. 10.1037/amp0000794. 33382294 PMC8299535

[smi70093-bib-0097] Zarrinabadi, N. , M. Rezazadeh , M. Karimi , and N. M. Lou . 2021. “Why Do Growth Mindsets Make You Feel Better About Learning and Your Selves? The Mediating Role of Adaptability.” Innovation in Language Learning and Teaching 16, no. 3: 249–264. 10.1080/17501229.2021.1962888.

[smi70093-bib-0098] Zeng, G. , X. Chen , H. Y. Cheung , and K. Peng . 2019. “Teachers ’ Growth Mindset and Work Engagement in the Chinese Educational Context : Well‐Being and Perseverance of Effort as Mediators.” Frontiers in Psychology 10, no. April: 1–10. 10.3389/fpsyg.2019.00839. 31057463 PMC6482247

[smi70093-bib-0099] Zhang, F. , and S. K. Parker . 2019. “Reorienting Job Crafting Research: A Hierarchical Structure of Job Crafting Concepts and Integrative Review.” Journal of Organizational Behavior 40, no. 2: 126–146. 10.1002/job.2332.

[smi70093-bib-0100] Zhao, S. , Y. Zhang , C. Yu , et al. 2023. “Trajectories of Perceived Stress Among Students in Transition to College: Mindset Antecedents and Adjustment Outcomes.” Journal of Youth and Adolescence 52, no. 9: 1873–1886. 10.1007/s10964-023-01788-5. 37296270 PMC10255944

[smi70093-bib-0101] Zhao, Y. , G. Niu , H. Hou , et al. 2018. “From Growth Mindset to Grit in Chinese Schools: The Mediating Roles of Learning Motivations.” Frontiers in Psychology 9, no. OCT: 2007. 10.3389/fpsyg.2018.02007. 30405492 PMC6200841

