# Supplementary material for: Leveraging a Dual‐Focused Growth Mindset to Boost Employee Resilience and Work Well‐Being: Evidence From a Two‐Wave Survey and an Intervention Study
Source: Stress Health. 2025 Jul 29;41(4):e70093. doi: 10.1002/smi.70093 (PMC12306150; doi:10.1002/smi.70093)
Supplement: Supplementary file 1 — Supporting Information S1 [file SMI-41-e70093-s001.docx]

**Table A.** Results of confirmatory factor analyses **(Study 1)**

|  | | | | | | | |
| --- | --- | --- | --- | --- | --- | --- | --- |
| Models | χ2 | df | RMSEA | CFI | TLI | SRMR | compare mode differences |
| **1. Six-factor model*** | 917.79 | 438 | 0.04 | 0.93 | 0.92 | 0.05 |  |
| 3. Five-factor model | 1025.36 | 451 | 0.05 | 0.92 | 0.90 | 0.06 | Model 1 vs Model 3: Δχ2 (13) = 107.57, *p* < .001 |
| 4. Four-factor model | 1171.14 | 451 | 0.05 | 0.89 | 0.88 | 0.07 | Model 1 vs Model 4: Δχ2 (13) = 253.35, *p* < .001 |
| 6. Three-factor model | 1131.42 | 456 | 0.06 | 0.87 | 0.86 | 0.07 | Model 1 vs Model 6: Δχ2 (18) = 231.63, *p* < .001 |
| 7. Two-factor model | 1391.76 | 460 | 0.06 | 0.87 | 0.85 | 0.07 | Model 1 vs Model 7: Δχ2 (22) = 473.97, *p* < .001 |
| Note: **Six-factor model** includes: two dimensions of growth mindset, resilience, perceived work stress and two dimensions of work well-being; **Five-factor model** includes: collapsing two dimensions of work well-being into one factor; **Four-factor model** includes: collapsing two dimensions of growth mindset into one factor; **Three-factor model** includes: collapsing two dimensions of growth mindset and resilience into one factor; **Two-factor model** includes: collapsing two dimensions of growth mindset and resilience into one factor, and two dimensions of work well-being into one factor. * refers to the best fitting model. | | | | | | | |

**Table B.** The concept clarity among work-growth mindset, job crafting self-efficacy, and job crafting competence.

| Concept | Definition | Key Focus | Nature |
| --- | --- | --- | --- |
| Work-Growth Mindset | The belief that one can be agentic in changing and optimizing work conditions, such as tasks and relationships. | Belief in one’s capacity for proactive change at work | A broad motivational belief about one’s ability to improve work conditions |
| Job Crafting Self-Efficacy | The belief in one’s capability to modify job demands and resources to better fit personal needs (Roczniewska et al., 2020). | Confidence in one’s ability to engage in job crafting behaviors | A specific self-belief about executing job crafting successfully |
| Job Crafting Competencies | The knowledge, skills, and abilities necessary to achieve personal objectives through job crafting (Bruning & Campion, 2022). | Actual skills and competencies required to craft one’s job effectively | A set of tangible abilities enabling job crafting |
